# Auditory Figure-Ground Segregation Is Impaired by High Visual Load

**DOI:** 10.1523/JNEUROSCI.2518-18.2018

**Published:** 2019-02-27

**Authors:** Katharine Molloy, Nilli Lavie, Maria Chait

**Affiliations:** ^1^Institute of Cognitive Neuroscience, University College London, London WC1N 3AR, United Kingdom, and; ^2^Ear Institute, University College London, London WC1X 8EE, United Kingdom

**Keywords:** attention, auditory scene analysis, load theory, magnetoencephalography, MEG, multisensory

## Abstract

Figure-ground segregation is fundamental to listening in complex acoustic environments. An ongoing debate pertains to whether segregation requires attention or is “automatic” and preattentive. In this magnetoencephalography study, we tested a prediction derived from load theory of attention (e.g., Lavie, 1995) that segregation requires attention but can benefit from the automatic allocation of any “leftover” capacity under low load. Complex auditory scenes were modeled with stochastic figure-ground stimuli (Teki et al., 2013), which occasionally contained repeated frequency component “figures.” Naive human participants (both sexes) passively listened to these signals while performing a visual attention task of either low or high load. While clear figure-related neural responses were observed under conditions of low load, high visual load substantially reduced the neural response to the figure in auditory cortex (planum temporale, Heschl's gyrus). We conclude that fundamental figure-ground segregation in hearing is not automatic but draws on resources that are shared across vision and audition.

**SIGNIFICANCE STATEMENT** This work resolves a long-standing question of whether figure-ground segregation, a fundamental process of auditory scene analysis, requires attention or is underpinned by automatic, encapsulated computations. Task-irrelevant sounds were presented during performance of a visual search task. We revealed a clear magnetoencephalography neural signature of figure-ground segregation in conditions of low visual load, which was substantially reduced in conditions of high visual load. This demonstrates that, although attention does not need to be actively allocated to sound for auditory segregation to occur, segregation depends on shared computational resources across vision and hearing. The findings further highlight that visual load can impair the computational capacity of the auditory system, even when it does not simply dampen auditory responses as a whole.

## Introduction

Figure-ground segregation, the process by which an auditory object is perceptually extracted from the aggregate sound input, underlies key aspects of listeners' ability to make sense of the acoustic environment, including recognizing individual sounds within crowded scenes and understanding speech in noise. Whether segregation depends on attention has been a long-standing question in hearing research (Shamma and Micheyl, 2010; Shamma et al., 2011; Snyder et al., 2012; Puvvada and Simon, 2017), but despite decades of debate, the answer has remained elusive.

Most previous work has focused on the effect of top-down allocation of attention on segregation. Typically, this is explored using a contrast between conditions where participants intentionally listen for an auditory object (“focused attention”), and conditions of either passive listening (Snyder et al., 2006; Gutschalk et al., 2008; Thompson et al., 2011; O'Sullivan et al., 2015) or where top-down attention is allocated to a different stimulus (Carlyon et al., 2001; Cusack et al., 2004; Bidet-Caulet et al., 2007; Lipp et al., 2010; Billig and Carlyon, 2016). Although it is accepted that auditory segregation can improve with top-down allocation of attention, it remains unclear whether attention is necessary for segregation. Some studies concluded that focused attention is required (e.g., Carlyon et al., 2001; Gutschalk et al., 2008; Lu et al., 2017), whereas others showed that sophisticated scene analysis can occur even when attention is directed previously (Macken et al., 2003; Teki et al., 2011, 2016).

A resolution to this disparity may be provided by perceptual load theory, which models perception as a limited capacity process, with automatic allocation of processing resources to all stimuli within capacity (Lavie, 2005), including stimuli that are not part of the top-down task set. The theory predicts that resources are only fully withdrawn from task-irrelevant stimuli when a separate, explicitly attended task involves a sufficiently high level of perceptual load to exhaust all capacity. Thus, even when defined as unattended auditory input may be processed and segregated, due to “spillover” of resources during lower perceptual load.

To establish whether segregation requires attention, a load paradigm is required: auditory stimuli should be presented as task-irrelevant while participants explicitly attend a separate, well-controlled task that varies availability of resources for auditory processing through a manipulation of attentional load (Lavie, 1995, 2005; Lavie et al., 2014). A process that does not depend on the availability of general computational capacity will occur regardless of the attended task load, while a process which requires resources will suffer as capacity is depleted. Here we use this approach to understand how auditory scene analysis is affected by the degree to which a concurrent visual task loads resources.

**Figure 1.**
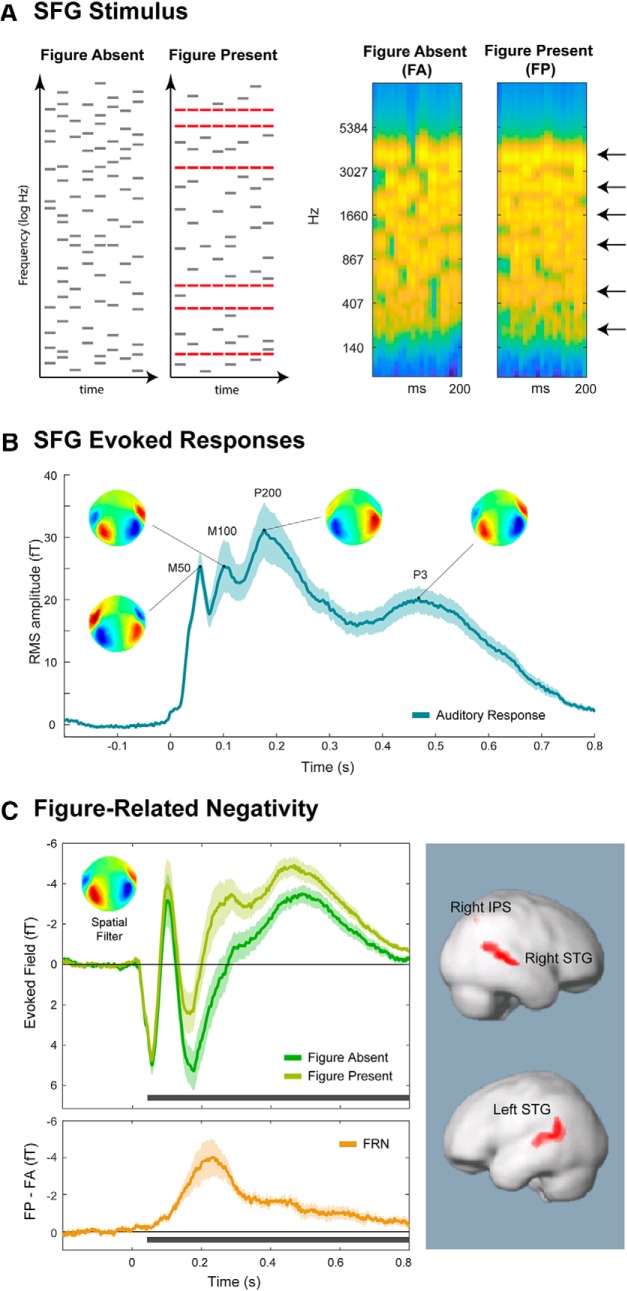
Stimuli and basic MEG responses under LL (Experiment 1). ***A***, Example schematics (left) and spectrograms (right) of the SFG stimuli. Stimuli, adapted from Teki et al. (2011, 2013), consisted of a succession of brief (25 ms) chords, each comprised of multiple frequency components. The FA stimuli were chords comprised of random frequencies, forming a stochastic background, whereas the FP stimuli were constrained so that a subset of the frequencies, selected randomly for each trial (indicated in red in the schematic representation and by black arrows in the spectrogram), were repeated across chords, producing an auditory “figure.” The associated percept is that of a bound auditory object that is segregated from the random ground (Teki et al., 2013). Stimulus examples are given in Figure 1-1. The spectrograms were generated with a filterbank of 1/equivalent rectangular bandwidth wide channels (Moore and Glasberg, 1983) equally spaced on a scale of equivalent rectangular bandwidth rate. Channels are smoothed to obtain a temporal resolution similar to the equivalent rectangular duration (Plack and Moore, 1990). This model processing in the auditory periphery produces a representation of the stimulus similar to that available to the CNS. ***B***, The overall response to the SFG stimuli (collapsed over FA/FP conditions) under LL (Experiment 1). Plotted is the mean RMS (instantaneous power) of stimulus-evoked activity collapsed over all conditions. Associated scalp topographies at major peaks are provided. Error bars indicate the Standard deviation of bootstrap resamplings. ***C***, FRN (Experiment 1). Evoked fields calculated using a spatial filter, which maximized the difference between FP and FA responses (inset; see Materials and Methods). Evoked fields in this and subsequent figures are plotted with M100 as an “upward” peak, to match the convention used in Electrocencephalography for its counterpart, N1. Error bars indicate the SD over bootstrap resamplings for each condition. Bottom, Horizontal black bars represent significant differences between the conditions. Top, Evoked responses separately for FP and FA stimuli. Bottom, FRN, calculated by the difference time-series of FP − FA. Right, Source-level contrast. Red represents regions where FP trials showed greater activity than FA trials. No regions were found to be significant for the opposite contrast (FA > FP).

To model complex auditory segregation, we used the stochastic figure-ground stimulus (SFG) (Teki et al., 2011, 2013, 2016) (Fig. 1). Similarly to natural sound mixtures (e.g., speech in noise), the SFG figure is not dissociable from the background based on instantaneous cues and can only be identified by integrating information over both frequency and time. Segregation of SFG signals is believed to occur via a process that detects correlations (“temporal coherence”) between individual frequency channels in auditory cortex (Shamma et al., 2011; Teki et al., 2013; O'Sullivan et al., 2015), demonstrated to play a vital role in segregating the spectrally broad, dynamic sounds experienced in natural environments. Accumulating work shows that listeners are sensitive to SFG figures, and brain responses to the emergence of the figure are consistently observed in naive listeners performing an incidental task (Teki et al., 2011, 2016; O'Sullivan et al., 2015). To determine whether the process underlying SFG segregation requires attention, we recorded magnetoencephalography (MEG) brain responses to task-irrelevant SFG stimuli while listeners performed a high load (HL) or low load (LL) visual task. Our results reveal that the brain response to the SFG figure was substantially reduced under visual load, highlighting that even basic auditory scene analysis draws on capacity, which is shared across the visual and auditory senses.

## Materials and Methods

### Experiment 1: MEG brain responses to short SFG sequences

#### Participants

Sixteen paid participants (9 male; mean age of 24.8 years, SD = 3.0 years) took part in Experiment 1. All were right-handed, had normal or corrected-to-normal vision, and reported normal hearing and no history of neurological disorders. The experimental protocol for all reported experiments was approved by the University College London research ethics committee.

#### Experimental design and statistical analysis

##### Apparatus, stimuli, and procedure.

The magnetic signals were recorded using a CTF-275 MEG system (axial gradiometers, 274 channels, 30 reference channels, VSM MedTech) in a magnetically shielded room. Participants were seated in an upright position, with the visual stimuli projected onto a screen placed ∼52 cm from the participants' eyes. Data were recorded continuously with a 600 Hz sampling rate and a 100 Hz hardware low-pass filter.

The auditory stimuli were ∼200-ms-long, diotically presented SFG stimuli (Teki et al., 2011, 2013, 2016). Signals consisted of a succession of chords, each comprised of multiple frequency components. Frequencies were chosen from a log-distributed pool of 109 frequencies from 180 to 4067 Hz. Each chord was comprised of between 11 and 21 (number was uniformly distributed) frequency components, which were selected from the frequency pool with equal probability. The “figure absent” (FA) stimuli (50%) were formed of random frequency chords. The “figure present” (FP) stimuli (50%) were constrained so that a subset of the frequencies were repeated in each chord (this parameter is referred to as the “coherence” of the figure) (Teki et al., 2011, 2013, 2016), whereas the others were selected randomly for each chord. The repetition of the coherent frequencies creates the auditory “figure,” which can be heard separately from the stochastically changing background (Teki et al., 2013). Figure 1*A* illustrates example FA and FP stimuli; sample sound files are available in Fig. 1-1.

The stimuli were varied along several parameters so as to optimize the stimuli to be used for the investigation of load (Experiment 2). Specifically, we varied the number of coherent frequencies used for the figure (6 or 8); the duration of the chords (25 or 30 ms); and the number of chords (6 or 8); all combinations were tested (8 possibilities), and stimuli were either FP or FA, creating 16 conditions. A total of 120 exemplars of each of the 16 conditions were randomly allocated into 4 blocks of 480 stimuli and presented with interstimulus intervals of 800 ms. Naive participants passively listened to the signals while performing an incidental visual task. Before the recording, the volume of the stimuli was set to a comfortable level (∼70 dB SPL) by each participant.

The visual task was designed to be very low demand. Pictures of landscapes were presented in groups of three (5 s per image, fade in and out over 1 s), and participants had to press a button if picture 2 or 3 was the same as picture 1. Instances of repetitions were relatively rare (∼1 in 12 sets) so that motor responses were kept to a minimum. This task helped ensure that participants' eyes were open, and they were awake throughout the blocks but did not place a high demand on processing resources.

At the beginning of the session, a short (4 min) “localizer” block was recorded to characterize participants' neural responses to simple auditory stimuli. The measurement consisted of 200 presentations of a 100-ms-long, 1 kHz pure tone with interstimulus intervals randomly distributed between 700 and 1500 ms. Participants watched a static fixation cross in the center of the screen and were not required to perform a task.

##### Analysis.

All conditions (over coherence, chord duration, and number of chords) showed very similar evoked responses; the data were therefore collapsed over all conditions for display purposes in the results, and for the subsequent source analysis.

The data from the localizer block were divided into 800 ms epochs and baseline-corrected using a 200 ms prestimulus interval. The M100 onset response (Roberts et al., 2000) was identified for each subject as a source/sink pair in the magnetic-field contour plots distributed over the temporal region of each hemisphere. For each subject, the 40 most activated channels at the peak of the M100 (20 in each hemisphere) were selected for subsequent sensor-level analysis of the responses evoked by the SFG stimuli.

The data from the main blocks were epoched into 1000 ms trials, which covered 800 ms after stimulus onset, and 200 ms before onset. All data were baseline-corrected to the pre-onset interval. Epochs with amplitudes >3 pT (∼6% of trials) were considered to contain artifacts and discarded. A PCA-based, denoising source separation (DSS) (de Cheveigné and Parra, 2014) routine was applied to the data to extract stimulus-locked activity. The 20 most repeatable components were retained and projected back to sensor space. To characterize the response at this stage, the root mean square (RMS) of the evoked field over the localizer channels was calculated for each time point to give a time-series, which reflects the instantaneous power of the evoked response. For illustrative purposes, group RMS (RMS of individual subject RMSs) is plotted (Fig. 1*B*), but statistical analysis was performed across subjects.

To characterize the elements of the response that are specific to FP stimuli, a further DSS was conducted, this time optimized to find components (spatial filters), which differed maximally between FA and FP trials (de Cheveigné and Parra, 2014). The highest ranked DSS component was retained for the analysis and used as a spatial filter (source model) for the analysis of FA versus FP trials (Fig. 1*C*). In all cases, this spatial filter corresponded to the standard temporal dipolar pattern associated with auditory responses.

FP trials were characterized by increased negativity relative to FA trials. To quantify this effect, the difference between the evoked responses for FP and FA trials was calculated for each participant, and subjected to bootstrap resampling (1000 iterations, balanced) (Efron and Tibshirani, 1993). The difference was judged to be significant if the proportion of bootstrap iterations which fell above/below zero was >95% (i.e., *p* < 0.05) for ≥15 adjacent samples (25 ms). The bootstrap analysis was run over the entire epoch duration (200 ms pre-onset to 800 ms after onset); all significant intervals identified in this way are indicated in the figure.

Sources were estimated using multiple sparse priors (Litvak and Friston, 2008) analysis. Inversions were based on all MEG channels and used a single-shell head model with group constraints. Second-level analyses consisted of *t* contrasts to compare activation between FP and FA conditions. Results were thresholded at *p* < 0.001 at the peak level and then subjected to a whole-brain *p* < 0.05 FWE correction at the cluster level. In one instance, a small-volume correction (a 10-mm-diameter sphere centered at the peak of the cluster) was applied instead because the cluster was small but in a location consistent with previous fMRI (Teki et al., 2011) and MEG (Teki et al., 2016) sources for similar SFG stimuli. The use of a different correction is marked in the relevant Table.

### Experiment 2: effect of visual load on figure-ground segregation

#### Participants

Twenty paid participants (8 male; mean age of 24.5 years, SD = 4.3 years), none of whom had previously participated in Experiment 1, took part in Experiment 2. All were right-handed, had normal or corrected-to-normal vision, and reported normal hearing and no history of neurological disorders.

#### Experimental design and statistical analysis

##### Apparatus and stimuli.

The apparatus and recording methods were identical to those in Experiment 1.

A feature versus combination visual search task was used to implement different levels of visual perceptual load (Treisman and Gelade, 1980; Lavie, 1995). The visual search arrays, presented for 200 ms on a dark gray background, consisted of five colored shapes spaced equally around a (nonvisible) circle centered at fixation and subtending 1.9° viewing angle. The five shapes comprised one each of a circle, triangle, square, diamond, and pentagon. The colors were assigned so that there were always two red items, two green, and one either blue or yellow (50% of trials each). In principle, any display could be used as an LL (color search) or HL (color-shape combination search) stimulus, so that displays were identical between load conditions. The target in LL was any blue shape; the HL targets were a red circle or green square. Targets were present in 50% of arrays and counterbalanced so that LL and HL targets did not correlate (i.e., if the LL target was present in an array, the likelihood of the HL target being present was 50%, and vice versa). The positions of the shapes were pseudo-randomized on each trial so that the target had an equal probability of occurring in each position. Each combination of shape and color was equiprobable across the stimulus array sets.

**Figure 2.**
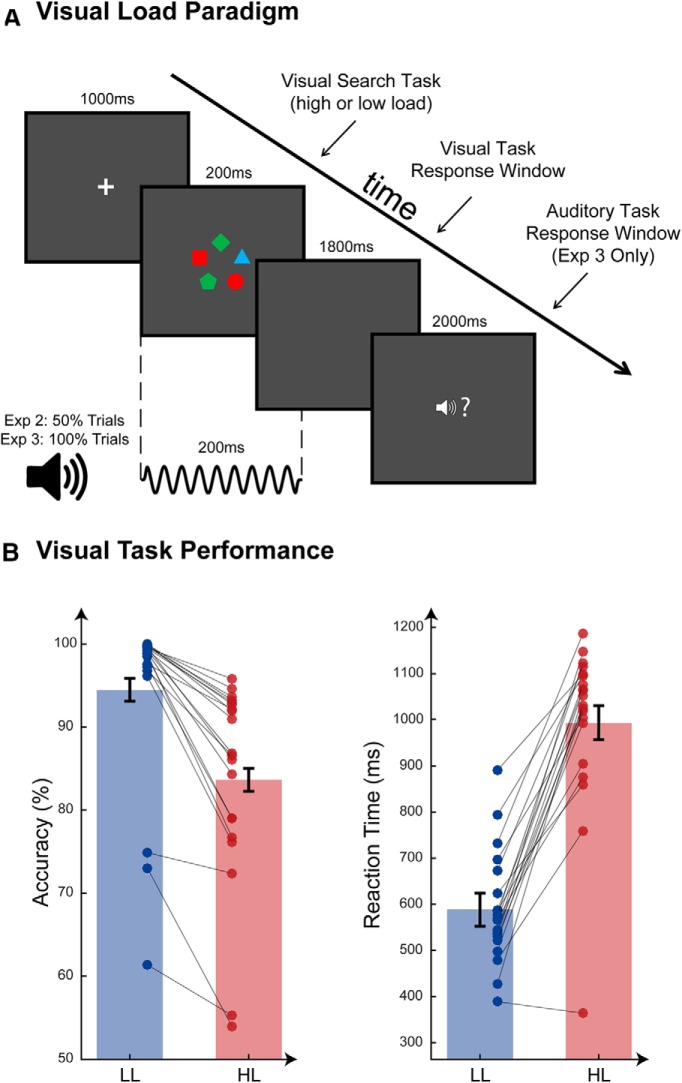
Experiment 2: visual load task. ***A***, Load task paradigm used in Experiments 2 and 3. The LL task was a color feature search, whereas the HL task was a color-shape combination search. Auditory stimuli occurred on 50% of trials in Experiment 2 (MEG) and 100% of trials in Experiment 3 (behavioral dual task). When present, auditory stimuli occurred at precisely the same time as the visual search array. The response window for the auditory target was displayed only during Experiment 3 when there was an active auditory task. ***B***, Visual task behavioral data from Experiment 2 (MEG). Mean values for accuracy (percentage correct) and RTs are plotted for LL (blue bars) and HL (red bars). Error bars indicate the Standard Error of the sample mean, corrected to reflect the within-subjects contrast. Individual data are plotted and connected by gray lines to illustrate change in performance for each participant between LL and HL conditions.

On half of the trials, the visual display was accompanied by a brief auditory stimulus, presented at the same time and for the same duration as the display (Fig. 2*A*). The auditory stimuli were identical to the SFG stimuli used in Experiment 1, but with fixed parameters: coherence 6, chord duration 25 ms, and chord number 8, producing a 200-ms-long stimulus (FP and FA with equal probability). There was no active auditory task; participants were encouraged to focus on the visual task and were told that the sounds were incidental.

The experiment consisted of 12 blocks (6 LL, 6 HL) consisting of 80 trials each; the order of blocks (low or HL) was counterbalanced between participants. Trial-by-trial feedback was not given, but at the end of each block, participants were provided a score of percentage correct on the visual task, to boost engagement. Blocks lasted for ∼4 min each, and participants were encouraged to take breaks between blocks when needed.

##### Procedure.

Figure 2 shows a schematic diagram of the trial structure. Each trial began with a fixation cross presented at the center of the screen for 1000 ms. Subsequently, a visual search array was presented for 200 ms, accompanied on 50% of trials by an auditory stimulus. A blank screen was then presented for 1800 ms, during which participants were to make a speeded response as to whether the visual target was present or absent (by pressing one of two buttons with their right hand).

##### Analysis.

The behavioral data from the visual task were analyzed to compare means within subjects for LL versus HL blocks. For the reaction time (RT) data, a paired-samples *t* test was run. For the percentage correct data, performance was close to ceiling and thus not normally distributed, so a Wilcoxon Signed Ranks Test was performed instead of the paired *t* test.

The MEG data were epoched into 1000 ms trials, including a 200 ms pre-onset interval. All data were baseline-corrected to the pre-onset interval. Epochs with amplitudes >3 pT (∼6% of trials) were considered to contain artifacts and discarded. DSS (de Cheveigné and Parra, 2014) was applied to the data to extract stimulus-locked activity. As with the previous analysis, the 20 most repeatable components were retained.

**Figure 3.**
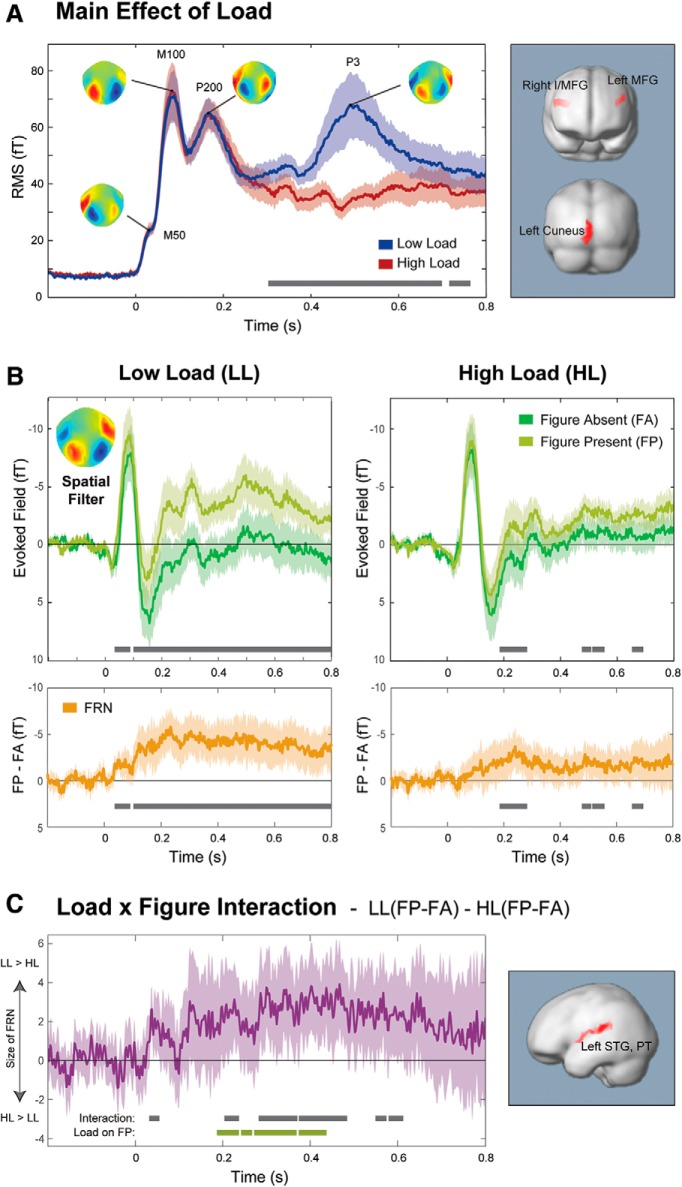
Experiment 2: effect of load on figure-ground segregation. ***A***, Overall response to the SFG stimuli (collapsed over FA/FP conditions) as a function of load. Mean RMS (instantaneous power) of responses to auditory stimuli (collapsed over FP and FA trials) in Experiment 2 under LL and HL, with scalp maps of peak topographies. The topographies are characteristic of auditory activity (symmetric dipolar pattern over temporal sensors), confirming that the source separation analysis was successful at isolating auditory activity. Error bars indicate the SD of bootstrap iterations for each condition. Bottom, Horizontal black bars represent significant differences between the conditions. Right, Regions where activity was stronger under LL than HL. No regions were found to be significant in the opposite direction to that displayed. ***B***, FP/FA responses as a function of visual load. Evoked fields illustrating the FRN separately under LL (left) and HL (right) conditions. Top, Evoked responses separately for FP and FA stimuli. Bottom, FRN explicitly, calculated by the difference time-series of FP-FA. Inset, Spatial filter used to calculate the responses (see Materials and Methods). Error bars indicate the SD of bootstrap resamplings for each condition. Bottom, Horizontal black bars represent significant differences between the conditions. ***C***, Interaction between load and FP/FA. The difference time-series, LL(FP − FA) − HL(FP − FA), quantifies the interaction between load and figure. Error bars indicate 2 SD of the bootstrap, for comparison with zero line. Black bars represent periods when the values differed significantly from zero. Green bars represent periods when load had a significant effect on responses to FP stimuli (no significant periods were found for the effect of load on FA responses). Right, Regions where the source analysis showed a significant interaction between responses in LL versus HL and FP versus FA.

Because the auditory stimuli were always presented concurrently with the visual search array, a further DSS step was necessary to separate auditory responses from the measured auditory-visual combined response. This analysis (collapsed over load conditions) identified components in the data, which showed the greatest difference between trials when the visual stimuli were presented alone (50%) and those when an auditory stimulus was also present (50%), with the aim of isolating activity, which specifically relates to the auditory stimuli (de Cheveigné and Parra, 2014). Therefore, this analysis should in principle eliminate any differences between HL and LL conditions that are driven by visual processing. The 10 highest ranked components were projected back into channel space, and the dataset was split into the LL and HL conditions. The RMS and scalp topographies (Fig. 3*A*) of the auditory component calculated from this analysis closely resemble the data recorded in response to the same stimuli in Experiment 1 (Fig. 1*B*), demonstrating that the auditory evoked activity was successfully extracted.

As in Experiment 1, a subsequent DSS analysis was applied to produce a spatial filter, which reflects activity most different between FP and FA trials. For this analysis, data were collapsed over load conditions so as not to bias any effects. The data were then separated into LL/HL and FP/FA conditions for analysis (Fig. 3). Statistical analyses between conditions were performed via bootstrap as described for Experiment 1.

To assess the relationship between perceptual load and the process of figure-ground segregation, we ran a correlation analysis. For each individual, the decrement in visual task performance as load increased was quantified by subtracting the mean RT under LL from that under HL. The effect of load on the amplitude of the figure-related negativity (FRN) was also calculated for each individual, by subtracting the mean amplitude of the FRN between 50 and 600 ms in HL from that in LL. A Spearman's rank correlation analysis was used to assess the relationship between these two factors across subjects.

Source inversions were calculated using multiple sparse priors (Litvak and Friston, 2008) analysis. Inversions were based on all MEG channels and used a single-shell head model and group constraints. For estimating sources of auditory activity, a soft prior over temporal and parietal areas was used, motivated by previous fMRI and MEG data for SFG stimuli (Teki et al., 2011, 2016), and our source results from Experiment 1, all of which indicate potential sources throughout the temporal lobe and in intraparietal sulcus (IPS). The prior mask was created in FSLview (http://surfer.nmr.mgh.harvard.edu/), based on combining the Harvard-Oxford Structural atlases for all temporal areas, and the Juelich histologic atlas for IPS, with a threshold of 5%. This resulted in a very broad prior, which was binarized so that the strength was equal over all regions. Solutions were not restricted to this mask; it served only as a before the source algorithm.

Second-level analyses consisted of paired *t* contrasts to compare the visual and auditory responses between LL and HL, and a full factorial RM *F* contrast to model the auditory responses, including main effects of load and figure, and the load × figure interaction. Results were thresholded at *p* < 0.001 at the peak level and then subjected to a whole-brain *p* < 0.05 FWE correction at the cluster level.

### Experiment 3: psychophysics dual task

#### Participants

Thirteen paid participants, none of whom had taken part in either of the previous MEG experiments, took part in the behavioral study. One was excluded because of extremely poor performance on the low-load task (61%; average of all included participants was 97.5%). For the remaining 12 participants (8 female), ages ranged from 18 to 35 years (mean = 21.4 years, SD = 4.1 years). All participants had normal or corrected-to-normal vision and reported normal hearing.

#### Experimental design and statistical analysis

##### Apparatus and stimuli.

The experiment was run on a Dell PC with a 13 inch monitor using MATLAB 7.12 (The MathWorks) and Cogent 2000 (http://www.vislab.ucl.ac.uk/cogent.php). A viewing distance of 57 cm was maintained throughout using a chin rest. Sounds were presented via tubephones (E-A-RTONE 3A 10 Ω, Etymotic Research) inserted into the ear canal. The stimuli were identical to those used in Experiment 2.

##### Procedure.

Trials were similar to those in Experiment 2, except that the auditory stimuli were present on every trial, and participants were asked to perform a dual task, responding first to the visual search (target present or absent) and subsequently to the presence of an SFG “figure” (FP stimuli). Trials were similar to those in Experiment 2, but after the response to the visual search (using their right hand), a prompt was displayed on the screen for 2000 ms (see Fig. 2*A*) during which participants indicated whether they had heard the auditory figure by pressing a button with their left hand. The experiment consisted of 12 blocks of 40 trials each, 6 LL and 6 HL, with the order of blocks counterbalanced between participants.

The experimental session was preceded by a series of short demo blocks (with trial-by-trial feedback), which introduced the auditory and visual tasks separately, and then combined them to illustrate the procedure for the dual task.

##### Analysis.

The data for both tasks were analyzed to compare means within subjects for LL versus HL blocks. For the visual task, a paired-samples *t* test was run on the RT data while a Wilcoxon Signed Ranks Test was performed on the percentage correct data, due to near-ceiling performance in LL. For the auditory task, hit rate, *d′*, false alarm rate and criterion (β) were calculated and subjected to paired sample *t* tests.

## Results

### Experiment 1: MEG brain responses to short SFG sequences

In this series of experiments, we focus on brief (∼200-ms-long) SFG bursts that occasionally (in 50% of the trials) contain a figure (Fig. 1*A*). As a first step, Experiment 1 was designed to characterize the MEG response under passive listening conditions. To maintain vigilance, participants were engaged by a simple, very LL, incidental visual task.

10.1523/JNEUROSCI.2518-18.2018.f1-1 Figure 1-1 Stimulus examples (.zip file containing stimulus examples and descriptions). Download Figure 1-1, ZIP file

Figure 1*B* shows the evoked response to the SFG stimulus, collapsed over FP and FA trials. Because these data are comprised of 20 PCA-like (DSS) components (i.e., reflect the independent activity of many processes), their dynamics are summarized by calculating instantaneous power (RMS; see Materials and Methods) across channels. Visible is the characteristic succession of onset peaks (M50, M100, P200 at 50, 100 and 200 ms after onset), followed by a P3 response from ∼300–700 ms after stimulus onset.

A source separation analysis (see Materials and Methods) was used to identify neural activity that is most different between FP and FA trials. The associated spatial filter (in the inset) was applied to the data to produce the time series in Figure 1*C*. The response to the FP trials relative to FA trials is characterized by a sustained “negativity” (i.e., in the same direction as the M100 peak). This effect, which we refer to as the FRN, is illustrated by the difference time-series (FP − FA), plotted at the bottom of Figure 1*C*. The FRN is apparent throughout the response, emerging as significant from 43 ms after stimulus onset and peaking at ∼200 ms after offset. The pattern is generally reminiscent of the object-related negativity (ORN) response, which has been observed when simultaneous auditory stimuli are perceived as two objects rather than one (usually a mistuned harmonic within an otherwise harmonic chord) (Alain et al., 2001; Alain and Izenberg, 2003; McDonald and Alain, 2005; Alain and McDonald, 2007), and more recently also in figure-ground stimuli similar to those used here (Tóth et al., 2016). The ORN is typically superimposed on the N1-P2 complex, peaks between 150 and 300 ms after stimulus onset, and can occur even when auditory stimuli are not actively attended.

These results confirm that there is a measurable neural response to the presence of the figure even during very brief SFG signals, consistent with previous behavioral reports (Teki et al., 2013) and despite the fact that the sounds were not explicitly attended. The fact that a response to the figure can be seen within 50 ms of scene onset (approximately two chords) suggests a very rapid, sensitive figure-ground segregation process.

Source localization revealed several brain regions where activity differed significantly between FP and FA trials (Fig. 1*C*; Table 1). FP stimuli showed greater activity in bilateral superior temporal gyri and right superior and inferior parietal lobules. This activity is consistent with the findings of Teki et al. (2011, 2016) that the SFG stimuli evoke figure-specific activity along the superior temporal planes, superior temporal sulci, and also within the intraparietal sulci.

**Table 1.** Experiment 1: effect of figure*^a^*

Cluster		Peaks			
Cortical structures	*p*(FWE-corr)	*t*	Coordinates (*x*, *y*, *z*)		
Left temporal lobe	<0.001	5.12	−58	−42	10
Superior temporal gyrus, BA 22		4.63	−58	−52	24
Right temporal lobe	<0.001	5.72	56	−42	10
Superior temporal gyrus, BA 22					
Right parietal lobe	0.026*^b^*	3.54	34	−66	46
Superior parietal lobule, inferior parietal lobule, BA 7		3.32	34	−62	48

*^a^*Source estimates for the difference between FP and FA trials.

*^b^*Small-volume correction.

### Experiment 2: effect of perceptual load on figure-ground segregation

Unlike many stimuli that are commonly used to study auditory segregation (e.g., the ubiquitous streaming paradigm or the informational masking paradigm) (Gutschalk et al., 2008; Elhilali et al., 2009a), SFG figures are not distinct from the background. Components belonging to the “figure” and those that are part of the “ground” are spread across the spectrum such that, at any given point in time, the “figure” and the “ground” do not provide inherently separate signals within the tonotopically organized auditory processing hierarchy. The percept of a “figure” emerging from the background arises from processes (“temporal coherence”) (Elhilali et al., 2009b; Krishnan et al., 2014), which computationally segregate the figure from the ground by analyzing information over time (over consecutive chords) and frequency to identify the components that change together (Teki et al., 2013, 2016). To understand how these processes are affected by the availability of processing resources, we recorded MEG responses to nonattended SFG signals while attention was engaged by a concurrently presented visual task, which placed different levels of load on perceptual processing.

#### Visual task

A significant effect of load on performance in the visual task was observed (Figure 2*B*). Increased load led to lower accuracy (percentage correct: mean: LL = 94.5%, HL = 83.7%; SD: LL = 10.7, HL = 11.9; Wilcoxon Signed Ranks Test *Z* = −3.9, *p* < 0.001; *d′*: mean: LL = 4.3, HL = 2.5; SD: LL = 1.3, HL = 1.0; *t*_(19)_ = 9.9, *p* < 0.001) and longer RTs (mean: LL = 594 ms HL = 1000 ms; SD: LL = 128, HL = 184; *t*_(19)_ = −10.5, *p* < 0.001), confirming that the load manipulation was successful.

#### Effect of perceptual load on overall response to ignored sound

To establish whether perceptual load had an effect on the base response to auditory stimuli (i.e., independently of whether a figure was present or absent), auditory components of the evoked response (after separation from visual evoked activity, but before isolating the figure response; see Materials and Methods) were calculated. The responses (collapsed over FP and FA conditions) are illustrated in Figure 3*A* using the RMS over channels. The activity is characterized by the standard succession of auditory response peaks, and the field maps associated with the major peaks (also plotted) exhibit the standard dipolar pattern over temporal channels commonly associated with auditory activity. The data closely match the responses observed during passive listening (Experiment 1; Fig. 1*B*), confirming that the auditory activity was isolated successfully from the response mixtures.

Comparing responses under low and high visual perceptual load revealed significant effects of load from 303 ms after stimulus, with a clear P3 “awareness” response apparent in the responses to the sounds under low, but not high, visual load.

Source analysis revealed significantly stronger activity in frontal and occipital regions in LL compared with HL (Fig. 3*C*; Table 2). The activity in bilateral middle frontal gyri is likely to be the source of the P3 response, which was apparent under LL, but not HL; the P3 has reliably been shown to have a generator in the frontal lobe when it occurs in response to nontarget stimuli (Comerchero and Polich, 1999; Simons et al., 2001; Goldstein et al., 2002; Polich, 2007; Shen et al., 2018). The difference in activity within the left occipital lobe may indicate some residual visual activity.

**Table 2.** Experiment 2: main effect of load*^a^*

Cluster		Peaks			
Cortical structures	*p*(FWE-corr)	*t*	Coordinates (*x*, *y*, *z*)		
Left occipital lobe	0.001	4.46	−4	−92	−12
Cuneus, precuneus, inferior occipital gyrus, BA 17, BA 18		3.92	−6	−84	18
		3.70	−2	−100	2
Right frontal lobe	0.025	3.96	48	16	30
Middle frontal gyrus, inferior frontal gyrus		3.94	44	20	22
Left frontal lobe	0.022	4.46	−48	20	30
Middle frontal gyrus					

*^a^*Source estimates for the difference between auditory responses in low and high load trials (regardless of figure presence).

#### Effect of perceptual load on figure-ground segregation

Our main focus is on the differences between FP and FA trials: Figure 3*B* illustrates the auditory evoked responses under LL and HL. The FRN for each load condition is shown underneath the main plots. Overall, the responses in this experiment, even under LL, are somewhat noisier than those in Experiment 1 (Fig. 2). This is likely due to the substantially lower number of trials for each condition, necessary to accommodate the load manipulation, and the fact that the auditory responses had been isolated from within the aggregate auditory-visual response. Importantly, a clear FRN was nonetheless observed. Under LL, the FRN was significant from 33 ms after onset until the end of the epoch. In contrast, under HL, the FRN only became significant at 185 ms after stimulus onset and was apparent during several shorter intervals late in the trial (see Fig. 3*B*). This difference suggests that high visual load substantially reduced the auditory system's ability to distinguish between FP and FA scenes. This was confirmed explicitly by evaluating the interaction between FP/FA and HH/LL conditions: for each subject, a difference time-series (LL (FP − FA) − HL (FP − FA)) was computed and subjected to bootstrap resampling. Figure 3*C* plots the resulting mean difference across subjects, confirming a significant effect of load on the FRN from ∼50 to 75 ms after onset (i.e., during the onset of the FRN) and from ∼200 ms after onset thereafter (i.e., during the peak of the FRN and onwards).

To understand whether this effect was driven by a load effect on FP trials, FA trials, or both, we compared HL and LL responses for FP and FA stimuli separately. This analysis demonstrated that load only had a significant effect on FP responses (Fig. 3*C*, green horizontal lines). Load did not have a significant effect on responses to FA stimuli.

Overall, these data indicate that high visual perceptual load impacted auditory processing that was specifically related to detecting the figure (as opposed to reducing responses to sound overall). This included an effect on the early stages of processing and persisted throughout the evoked response.

To further assess the relationship between perceptual load and the process of figure-ground segregation, we calculated the correlation between the impact of perceptual load on individuals' RTs for the visual task and the impact of load on the amplitude of the FRN (see Materials and Methods). There was a significant positive correlation between the two measures (Spearman's *r_s_*(20) = 0.572, *p* < 0.01), indicating that the participants who showed larger performance deficits on the visual task as load was increased also showed more substantial reductions in the neural signature of auditory figure-ground segregation in the HL compared with LL conditions. This further supports the critical role of demand on processing resources in determining the magnitude of the cortical response to auditory figure-ground segregation.

#### Source analysis

When collapsed over LL and HL, source analysis identified areas in the right temporal and right parietal lobes, which showed greater activity in response to FP versus FA scenes (Table 3). The temporal region covered the posterior portion of the right superior temporal gyrus, with some extension to middle temporal gyrus and planum temporale (PT). This closely mirrors the bilateral temporal sources seen in Experiment 1, and previous fMRI and MEG data (Teki et al., 2011, 2016). The parietal source covered regions of the superior and inferior parietal lobules. It was slightly superior and anterior to the source seen in Experiment 1, and overall more diffuse, but given the relatively poor spatial resolution for MEG, we believe both represent activity within the IPS. Both these loci are also consistent with the fMRI and MEG data discussed above (Teki et al., 2011, 2016). This further confirms that the DSS analysis successfully captured the relevant SFG evoked activity.

**Table 3.** Experiment 2: main effect of figure*^a^*

Cluster		Peaks			
Cortical structures	*p*(FWE-corr)	*F*	Coordinates (*x*, *y*, *z*)		
Right parietal lobe	<0.001	21.64	48	−38	10
Inferior parietal lobule, superior parietal lobule, BA 40		20.74	66	−26	2
		20.66	52	−24	2
Right temporal lobe	<0.001	19.31	38	−38	44
Superior temporal gyrus, middle temporal gyrus, BA 22, BA 41, BA 42		17.11	42	−26	42
		16.74	38	−30	48

*^a^*Source estimates for the difference between auditory responses in FP and FA conditions (regardless of load).

The source of the interaction between load and figure was localized to the left temporal lobe (Fig. 3*B*; Table 4), an area that extended down the left superior temporal gyrus and PT, including Heschl's gyrus. This suggests that relatively early cortical processing of the SFG stimuli was affected, in keeping with the fact that the evoked data showed an early impact of load. The left-lateralized effect is commensurate with the main effect of figure described above, which was significant in the right, but not left, temporal gyrus.

**Table 4.** Experiment 2: figure × load interaction*^a^*

Cluster		Peaks			
Cortical structures	*p*(FWE-corr)	*F*	Coordinates (*x*, *y*, *z*)		
Left temporal lobe	0.006	25.82	−50	−30	18
Superior temporal gyrus, planum temporale BA 41, BA 42		14.69	−60	−2	6
		13.67	−62	−16	14

*^a^*Source estimates for the interaction between load (low, high) and figure presence (FP, FA).

### Experiment 3: psychophysics dual task

Experiment 3 was designed to determine whether the reduced figure-ground segregation that was shown in Experiment 2 under high (relative to low) visual load was associated with a reduction in perception of the auditory figures. This experiment used a behavioral dual-task design to evaluate whether high visual load could impair detection of the auditory figures, even when participants were actively listening for them. We combined the previous visual task with a secondary auditory figure detection task, which participants performed concurrently.

Results are shown in Table 5. Similarly to the behavioral pattern in Experiment 2, participants showed a significant effect of load on performance in the primary, visual, task. Increased load led to lower accuracy (Wilcoxon Signed Ranks Test *Z* = −3.1, *p* < 0.011) and longer RTs (*t*_(1,11)_ = −12.5, *p* < 0.001), indicating that the load manipulation was successful. Higher load in the primary visual task also led to poorer performance in the secondary, auditory, task: hit rates were reduced (*t*_(1,11)_ = 4.2, *p* = 0.001), and participants showed poorer sensitivity to the auditory target: *d′* scores were significantly reduced in the high compared with the LL condition (*t*_(1,11)_ = 3.7, *p* = 0.004), with no change in decision criterion (β) or false alarm rates.

**Table 5.** Experiment 3: dual task behavioral data

	Auditory task								Visual task			
	*d′*		Beta		FA rate		Hit rate		% correct		RT (ms)	
	LL	HL	LL	HL	LL	HL	LL	HL	LL	HL	LL	HL
Mean	1.8	1.5	0.80	0.91	31.1	32.8	85.5	80.5	97.5	89.9	714	1066
SD	0.27	0.23	0.16	0.15	5.7	5.9	3.3	3.6	0.5	1.4	42	31
*p*	0.004		0.178		0.440		0.001		<0.01		<0.001	

The auditory detection performance was overall high, confirming that participants could successfully identify the figure within very short auditory scenes when these receive attentional resources in conditions of low perceptual load. However, crucially, HL reduced participants' ability to hear the auditory figures, even when they were actively listening for them. While the effect was relatively small, this was due to the necessary confound present in dual tasks whereby the “secondary” stimuli are still task-relevant and therefore attended, making any load manipulation much weaker. Thus, in the MEG studies (where sounds were fully ignored and participants were naive to the potential figures), we would expect a much more substantial effect on awareness, in line with the strong effects demonstrated on the FRN response.

## Discussion

Previous studies demonstrated that auditory segregation is improved with focused attention (Carlyon et al., 2001; Cusack et al., 2004; Snyder et al., 2006; Gutschalk et al., 2008; Lipp et al., 2010; Thompson et al., 2011; O'Sullivan et al., 2015). For example, Gutschalk et al. (2008) showed that brain responses evoked by a tone stream (“target”) embedded within a tone cloud were substantially enhanced when listeners actively attended to the target relative to when attention was directed to an unrelated stimulus in the other ear or during passive listening. These findings are consistent with an account according to which passive listening promotes a broad variety of stimulus processing to monitor all aspects of the environment, but when segregation becomes the focus of attention that specific process can be given priority.

In contrast, here we establish that figure-ground segregation within task-irrelevant sounds is affected by the degree to which a concurrent visual task loads resources. The results demonstrate that the critical issue is not whether top-down attention is allocated toward or away from sound, but rather the level of load on the perceptual system, revealing an extensive impact of visual perceptual load on auditory figure-ground segregation in task-irrelevant sounds. MEG recordings showed that low visual perceptual load was associated with a clear neural response to the auditory figures (the FRN). In contrast, conditions of HL resulted in a substantial FRN reduction. An additional behavioral experiment demonstrated that increased visual load led to poorer detection of auditory figures, even when participants were intently listening for them. Overall, these findings suggest that increased visual perceptual load can reduce the efficacy of the computations underlying figure-ground segregation, such that both the neural response to, and perceptual awareness of, the figure are impaired. Thus, while SFG segregation occurs by default when resources are available (see also Teki et al., 2011, 2016), it can fail when attention is diverted to a demanding, prioritized task, even one in the visual modality.

### Load theory

Load theory (e.g., Lavie, 2005, 2010) proposes that perceptual processing depends on limited resources that are allocated with higher priority to attended stimuli, but also involuntarily to task-irrelevant stimuli as long as these are within capacity. Thus, the degree to which task-irrelevant information is processed is determined by the level of perceptual load in the attended task. The present data support these predictions: in the passive listening (Experiment 1) and LL (Experiment 2) conditions, the auditory evoked fields reliably showed an FRN. However, conditions of high visual load resulted in a considerable reduction of the FRN. These results suggest that load theory, which was developed primarily within vision, is also a suitable model for processing in the auditory system (Murphy et al., 2017).

The critical effect of (visual) perceptual load on the FRN is further corroborated in the finding of a significant correlation between the impact of perceptual load on individuals' visual task RT and its impact on the amplitude of their FRN: larger load effects on RT (indicative of a greater impact on capacity) were associated with a larger load effect on the FRN. This is consistent with a recent report establishing individual differences in perceptual capacity (Eayrs and Lavie, 2018).

### Visual load can impair the computational capacity of the auditory system

Increased visual load can lead to a reduction of early cortical evoked responses to simple, low-amplitude, auditory stimuli (Dyson et al., 2005; Molloy et al., 2015). This can be interpreted as suggesting that high visual load can lower the “gain” on early auditory sensory representations. The present results suggest that withdrawing resources might also have a detrimental effect on the computational capacity of the auditory system. SFG signals provide a well-controlled test of computational capacity (as opposed to simple signal detection) because, at any single point in time, there is no distinction between figure and ground. To identify a figure, a process of spectrotemporal integration (by computing cross-channel temporal coherence) (Teki et al., 2013; O'Sullivan et al., 2015) must occur.

To specifically target the process of figure-ground segregation, we sought to minimize any general “gain reduction” effects of load by using loud SFG signals that had (as shown under LL) a robust, highly detectable figure. Indeed, the effects we observe demonstrate an impact of load specifically on figure-ground processing as opposed to reducing overall responses to sound: First, throughout the analyzed epoch, the effect of load was confined to the FP stimuli, whereas FA responses did not differ across load conditions. Second, the stark reduction of the FRN under HL was evident from as early as ∼50 ms after onset. This corresponds to the earliest portion of the FRN response as measured during passive listening (Experiment 1; Fig. 1*C*). While the FRN is present during this interval in the LL condition, it is entirely abolished under high visual load (also confirmed with the interaction analysis). Because 50 ms (2 stimulus chords) is the latency at which the figure becomes technically extractable, we interpret the early effect as indicating that load impaired the computations, which underlie initial stages of extraction of the figure from the background.

That the effect of load on the FRN persisted until ∼600 ms after onset suggests that load also impairs subsequent processes (e.g., those associated with awareness of the figure). Indeed, the later portion of the significant interval overlaps with the time-window associated with the P3 “awareness” response (Picton, 1992; Comerchero and Polich, 1999; Kok, 2001; Polich, 2007).

The FRN reported here is similar to those reported in previous studies using extended, temporally dynamic stimuli (Elhilali et al., 2009a; Teki et al., 2016). It is also generally reminiscent of the ORN (Alain et al., 2001, 2002; 2003; Tóth et al., 2016), a response elicited between 150 and 300 ms, reflecting segregation of a tone from a sound complex. However, in contrast to the present results, Alain and Izenberg (2003) and Dyson et al. (2005) reported no effect of attentional load on the ORN. This discrepancy may be either due to segregation based on instantaneous cues being less susceptible to depletion of computational resources than segregation based on temporal coherence (Micheyl and Oxenham, 2010) or due to the visual attention tasks used previously not involving a sufficiently high level of load to exhaust capacity. Indeed, the conditions of 1-back (vs 0-back) in an *n*-back task, or incongruent (vs congruent) attributes of a single stimulus, used in Dyson et al. (2005), are not typically considered HL.

Our paradigm, using very brief stimuli and where the auditory signals were precisely temporally aligned to the visual stimulus, assured high visual-auditory processing overlap. Future research using the same loading task to compare FRN and ORN should prove useful to understand the potential differences between FRN and ORN in their demand on computational resources.

### Brain mechanisms underlying SFG processing

Segregation of SFG stimuli is hypothesized to involve processing of temporal coherence through cross-channel correlation (Teki et al., 2011, 2016), supported by rapid adaptive processes in auditory cortex (Elhilali et al., 2009b; Krishnan et al., 2014; Lu et al., 2017). Recent work indicated that this rapid plasticity only takes place when animals are explicitly attending to the auditory signals (Lu et al., 2017). This does not tally with the human literature, where segregation based on temporal coherence is reliably observed during passive listening (Experiment 1 here) (Teki et al., 2011; O'Sullivan et al., 2015). Our data show that the limiting aspect is not active attention per se, but rather the availability of computational resources to the ignored stimuli. Thus, a disparity in the size of resource pools between human and animal models could lead to the apparent differences between the levels of attention required for these adaptive processes.

Human neuroimaging work has implicated PT and IPS in the process of detecting SFG “figures” (Teki et al., 2011, 2016). A similar network is seen in the present experiments during LL/passive listening conditions. Teki et al. (2016) suggest that PT operates as a hub for the process that computes the coherence maps, whereas IPS is involved in encoding the signal as consisting of several sources.

Interestingly, here load specifically impacted processing in temporal cortex: the effect of load on the FRN was localized to the upper bank of the left superior temporal gyrus, including the PT and Heschl's gyrus. Activity in this region was generally more pronounced during FP scenes compared with FA, but the distinction was less marked under HL. This is consistent with both the findings of attention-dependent adaptation in A1 (Lu et al., 2017) and with the hypothesized role of PT in computing the temporal coherence maps, which underlie segregation (Teki et al., 2016). Importantly, our data demonstrate that processing within these regions is not encapsulated but draws on domain-general resources, such that conditions of high demand in the visual modality can lead to the failure of fundamental aspects of auditory processing.
